# Tracing Personalized Health Curves during Infections

**DOI:** 10.1371/journal.pbio.1001158

**Published:** 2011-09-20

**Authors:** David S. Schneider

**Affiliations:** Department of Microbiology and Immunology, Stanford University, Stanford, California, United States of America

## Abstract

It is difficult to describe host–microbe interactions in a manner that deals well with both pathogens and mutualists. Perhaps a way can be found using an ecological definition of tolerance, where tolerance is defined as the dose response curve of health versus parasite load. To plot tolerance, individual infections are summarized by reporting the maximum parasite load and the minimum health for a population of infected individuals and the slope of the resulting curve defines the tolerance of the population. We can borrow this method of plotting health versus microbe load in a population and make it apply to individuals; instead of plotting just one point that summarizes an infection in an individual, we can plot the values at many time points over the course of an infection for one individual. This produces curves that trace the course of an infection through phase space rather than over a more typical timeline. These curves highlight relationships like recovery and point out bifurcations that are difficult to visualize with standard plotting techniques. Only nine archetypical curves are needed to describe most pathogenic and mutualistic host–microbe interactions. The technique holds promise as both a qualitative and quantitative approach to dissect host–microbe interactions of all kinds.

## Abstract 2

By concentrating on the relationship between health and microbe number over the course of infections, most pathogenic and mutualistic infections can be summarized by a small alphabet of curves, which has implications not only for basic research but for how we might treat patients.

When I get infected, I don’t think, “my TLRs and inflammasomes are activating!” As an infected patient, I worry about two things: “how sick am I going to get?” and “when am I going to get better?” Physicians and nurses understand these questions innately because it is their job to keep us from getting sicker and to bring us back to health. There is an unfortunate disconnect between these issues and the questions basic scientists study. Basic scientists are terrific at uncovering the fundamental mechanisms controlling the activation of immune responses, identifying the effectors that clear microbes, and determining how much pathology will be caused by an infection. However, it is difficult to move from these molecular markers to the emergent properties of health and recovery in a patient.

Here I discuss two frameworks for considering the questions “how sick will I get?” and “when will I get better?” The first is the idea of tolerance–the dose response curve of health with respect to microbe number in a host population. This concept is well established in the plant literature [1]–[5] and has crossed over recently to discussions about infections in animals [6]–[8]. The second, and focus of this perspective, is a discussion on how to take this concept of a health-by-microbe space as defined by tolerance curves in populations and apply this to individuals. This approach highlights parts of the infectious process that are understudied and provides a new quantitative approach for attacking this problem.

There are many other models that describe host–microbe interactions, ranging from discrete mathematical models to global theories, and they run the gamut from mathematically rigorous constructions to thought-provoking cartoons [6],[9]–[17]. Each model is useful for highlighting a different aspect of host–microbe interactions, but the purpose here is to discuss tolerance and its extensions.

## Summarizing Infections in Populations Using the Concept of Tolerance

How can we summarize an infection by reducing it to a small number of points that can be compared within an infected population? One way is to pick obvious landmarks from health or microbe timelines of the infection, such as the peak parasitemia or the lowest health. If we plot these values together on a health-by-microbe graph we produce a single point for a patient. By collecting many of these points we can create a scatter plot showing what happens when a population is exposed to this pathogen. Ecological immunologists have defined an elegant system for discussing such graphs in which the relationship between health and microbe numbers in a scatter plot is defined as tolerance [1],[2]–[8],[18]. Tolerance is the dose response curve for the system; it defines the slope of health to parasite load in a population.

The concept of tolerance can be used as a tool to dissect infections. By monitoring how the curve changes when host genetics or environments are altered we can learn about the factors contributing to a host–microbe interaction. Tolerance allows us to differentiate between physiological mechanisms that are mostly required to clear pathogens and distinguish these from mechanisms that impact our health. This second group of mechanisms tends to be discussed less than immune effectors. Tolerance promises to teach us how to tune a body’s response so that we prevent microbe growth while limiting the negative effects on health.

Tolerance is useful for studying populations, not individuals. A tolerance curve informs us that humans in general react in a certain manner to a given pathogen and we can plan our treatment accordingly. If a newly infected patient walks into a doctor’s office there are two barriers to using tolerance to treat that person as an individual. The first barrier is that we would have to let the patient reach peak parasitemia and minimum health to place them on the above tolerance curve, and by that point they would have likely suffered through the worst part of the infection; that is no help. What we learn from a tolerance curve depends upon the way we define it; if we use the maximal parasite load and minimal health to summarize infections, then we don’t learn anything about recovery. The second barrier is that even if we could gather these summary data, we can only place one point on the scatter plot for this patient. This might tell us that the patient is aberrant, if they fall far from the curve, but we can’t measure their tolerance because the patient represents one point on the curve. We aren’t measuring their response in a variety of states and therefore can’t generate a dose response curve. How can we apply this idea of health-by-microbe space to personalized medicine?

## Applying Health-by-Microbe Number Space to Individuals

**Figure 1:**
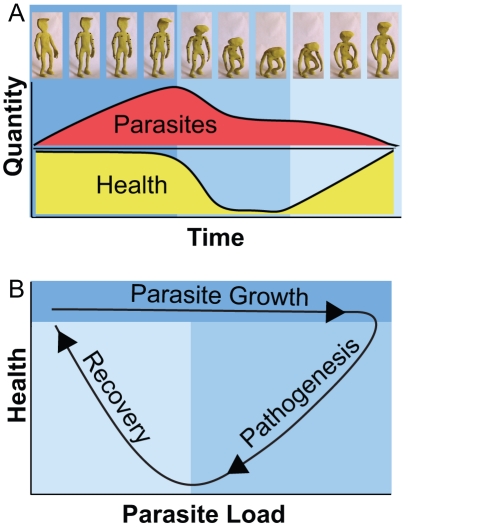
Plotting data in the phase plane to better monitor infections. (A) A sick “patient” is depicted in frames at the top where the red dots indicate parasites and the stature of the “patient” depicts health. In a simple timeline, parasites can be seen to rise and fall and the health falls and returns to its original levels. The relationship between health and parasite levels is visible but not as simple to interpret as shown below in (B). (B) The curves from (A) are replotted in a health by parasite load phase plot. The plot shows three sections: First, the parasites grow but do not affect health (dark blue). The slope here is quite flat. Second, (medium blue) the host begins to clear the pathogens but the health crashes as well in this pathogenesis portion of the plot. Third (light blue), the health recovers while the microbes continue to be cleared.

The conventional method of following infections is to plot dependent variables (for example: parasitemia, fever, anemia, weight loss) versus time; this obscures some important relationships (Figure 1A). For example, it is simple to pick out the peak values and times for health and parasitemia, but the relationship between health and parasitemia is harder to see because the relationship changes continuously. We presume that parasite load drives changes in health, but we seldom monitor this directly.

What would happen if instead of taking the peak parasitemia and minimum health as a summary of an infection, we plotted health-by-microbe values at every time point [11]? Imagine the individual depicted in Figure 1A. This patient is initially infected by a parasite, which produces a single large red lump on his hand. The parasite reproduces, creating more red lumps, but this doesn’t have a large effect on health. At some point the immune response turns on and the parasites are removed; the patient now suffers an immunity-driven loss of health, as indicated by his posture. Ultimately, the patient recovers his initial health and all of the parasites are cleared. This is a resilient system. By resilience, I mean the properties of a system that push it back to its original state following a perturbation. That we get better following an infection means that we are resilient.

Instead of plotting parameters versus time, I’ve plotted dependent parameters against each other in the phase plane as health-by-microbe number in Figure 1B. This produces a looping curve that better shows the relationship between health and microbe number across the whole infection. This relationship changes across the course of the infection: In the first portion, microbe load increases without affecting health. Next, both health and microbe numbers simultaneously crash. Finally, health increases and microbe numbers drop to zero.

Though all of the information is present in the original, this new type of plot reveals some properties that are hard to visualize from the timelines. It is clear that in this infection, microbes are not the direct cause of pathology; rather, the immune response is causing damage because there is no pathology until microbe clearance begins. The relationship that becomes very apparent out in this presentation is recovery; at some point during the infection the patient heals. Much of our research into microbial pathogenesis is directed towards limiting microbe growth or limiting pathology with the hope that if we don’t get severely ill then it will be easier to recover. This graphing approach highlights recovery and provides a quantitative method for measuring recovery.

**Figure 2:**
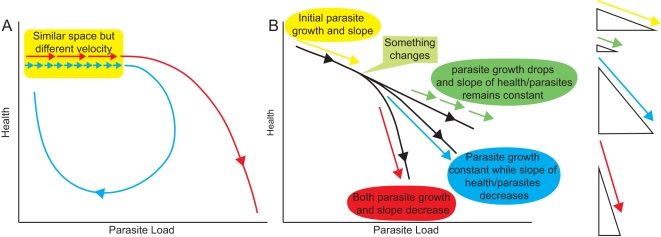
The contribution of velocity to disease curves. The cartoons in this article don’t show imaginary data points and thus don’t give an impression of the velocity that a host will pass through health-by-microbe space. Here I’ve used vectors to show velocity. (A) Depicts two curves, one resilient and another leading to parasite growth and host death. Near the origin, both curves traverse the same space and can’t be distinguished on this basis; however, the curves differ in velocity. This highlights the point that it is important to measure velocity when plotting these curves. (B) Depicts a bifurcation point in a curve after an unknown “something changes”. The three following curves differ in their velocity as indicated by the length and direction of the vector arrows. On the right, the vectors are compared next to triangles to make it easier to see the components controlling parasite growth and health. The green curve has exactly the same health to parasite slope as the original, but the velocity of the curve is reduced. Perhaps an antimicrobial has been induced that blocks parasite growth but does not harm the host. The blue curve has the same parasite growth rate but the slope is steeper. In this case an ineffective and host-damaging immune response could have turned on. The red curve shows a reduction in parasite growth and a decrease in slope. Here, an effective but host-damaging antimicrobial may have been produced. This figure highlights the importance of measuring the acceleration of these curves.

This presentation is useful as a two-dimensional map and it is easy to overlook the hidden third property–velocity. The spacing of each data point indicates how quickly an individual passes through health-by-microbe space (Figure 2A). For example, it is easy to imagine two individuals that traverse the same health-by-microbe space but differ in their velocity and that it is the velocity that leads to different outcomes. A change in velocity (acceleration) during the course of the infection in an individual also provides useful information (Figure 2B). For example, when the rate of parasite growth decelerates, that suggests that antimicrobial effectors are being produced. Likewise, when health starts to accelerate in a positive direction, this suggests that repair mechanisms are being expressed. It is therefore important to study the velocity and acceleration of these curves in addition to the simple phase space depiction of infection.

The disease curve shown in Figure 1B is drawn in two dimensions, but there is no theoretical limit to the number of dimensions that could be used. Physicians working in an intensive care unit might find this obvious as they monitor dozens of parameters when they coax a person’s health back to a survivable range. Those of us studying microbial pathogenesis in the lab tend not to look at all of these parameters at once, but by drawing even two-dimensional disease curves explicitly we can highlight processes that have been understudied.

## Applying the Idea of Health-by-Microbe Space Broadly to Infections

Having generated a disease curve from an imaginary infection, it is worthwhile to think about how these curves might look for well-studied infections. I suggest that there is a relatively small alphabet of curves that can describe most host–microbe interactions.

**Figure 3:**
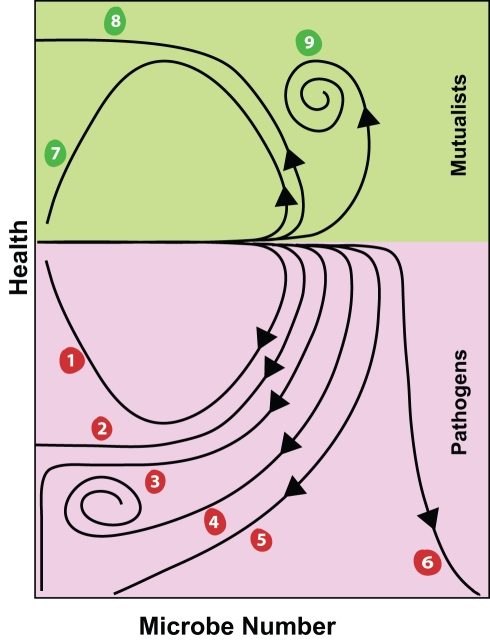
Nine simple curves describe the infectious route of all infections. Curve definitions: Pathogenic: 1. Recovery (uncomplicated flu, measles, gastritis). 2. Permanent and stable disability (lasting meningitis/encephalitis damage). 3. Unstable disability (rheumatic fever sequelae or reactive arthritis). 4. Persistent pathogen infection (tuberculosis, herpes). 5. Death while defeating a microbe (sepsis). 6. Uncontrolled microbial growth and death. Mutualistic: 7. Short-term colonization with a beneficial microbe (transient probiotics). 8. An infection that is cleared but permanently changes the state of the host (live vaccines). 9. Persistent infection with a mutualist (*Rhizobium*, *Hamiltonella*, *Wolbachia* [20]–[24], herpes [26]).

Regarding pathogens: In curve one (Figure 3), which could be used to describe an acute infection of uncomplicated dengue or flu, the pathogen levels rise, health falls, and both ultimately return to original healthy levels. Curve two depicts a situation where the host clears the pathogen but suffers irreversible damage, as might occur in a case of encephalitis or gangrene. Curve three shows a case where the microbe is cleared but the host becomes locked into an inflammatory state that causes further damage, triggering an autoimmune disease like reactive arthritis or rheumatic fever. The fourth curve shows a stable and non-resolving infection like tuberculosis (or see [19] for a related curve). The health placement of this whorl will vary with the particular infection; there are some situations, as with herpes or varicella infections where the steady state health of the host would be normal (or perhaps higher than the uninfected state as discussed below). The fifth curve shows the outcome of septicemic shock–like illnesses where the body is not failing to clear microbes, but the host dies because of overwhelming pathology. The final pathogenesis curve, six, depicts a situation where the host can’t control the growth of a microbe and this ultimately leads to death, for example, an uncontrolled gangrene or *Streptococcus pyogenes* infection.

These curves can also be used to describe mutualistic host–microbe interactions; this is critical because if new a system that strives to explain host–microbe interactions can’t describe mutualists as readily as it describes pathogens, then it is dead on arrival. Three of the disease curves described above can be inverted or rotated upwards to describe the interaction of hosts with beneficial microbes. There aren’t corresponding mutualist curves for each disease curve, as not all of the pathogenesis curves make sense when flipped. For example, a pathogenic infection that led to an unstable ever-decreasing health would be inverted to create a curve that led to constantly increasing health. This is a formula to create a superhero, which doesn’t happen often enough in modern medicine. Curve seven describes a fleeting interaction with a mutualist, perhaps a probiotic; the microbe provides benefit to the host while it is on its limited journey through the digestive track, and this benefit ends once host and microbe part ways. Curve eight describes a permanent change that could be induced in a host by a live vaccine that remains even after the attenuated pathogen has been removed. In this case it is likely that the initial infection would cause some pathology but would ultimately result in higher health. That higher health would be conditional, as it would depend upon later exposure to the infected pathogen. Curve nine describes a long-term mutualistic interaction that reaches a relatively steady state, like the association of humans with their gut microbiota or endosymbionts that can protect insects from infections by parasitoids or viruses [20]–[24].

All of the curves described above were drawn as strictly increasing or decreasing health, but there are some examples that could cross the line repeatedly. The bobtail squid–*Vibrio fischeri* symbiosis provides an example [25]. This squid has a light-producing organ that relies upon the bacterium *V. fischeri* to produce the light. These bacteria are harvested from seawater by the squid and are not passed down maternally. Every morning, the squid squirts out the majority of the bacteria in the light organ and then the organ regenerates and the bacteria grow back. This will produce a looping health-by-microbe curve that cycles every day. Herpes infections in mice, though they cause short-term decreases in health, can be protective against other infections [26]. This sort of curve would resemble the mutualist curve nine, except that it would initially dip below 100% health before it hit its final steady state.

There are cases where these sorts of phase curves will not be helpful in dissecting an infection. For example, if a parasite doesn’t replicate in the host, a phase curve of parasite number versus pathology will not be informative. An example of such a situation would be the pathology that hookworms or ascaris cause when the worms migrate through our bodies on the way to our guts, as this pathology doesn’t depend upon the replication of the parasites.

## Defining Microbe Levels

It should be simple to determine microbe levels for many pathogens. Insect-borne pathogens will be particularly easy because these have to reach relatively high levels in the circulation in order to be taken up by a blood-feeding insect. Plasmodia, trypanosomes, filarial worms, and arboviruses fit into this class since pathogen levels can be measured from the blood. Diarrhea-causing infections that shed microbes should be equally simple to assess. Infections of immune cells, like HIV, are also addressable in this fashion.

Theoretically, this approach will work for all infectious diseases because there is a relationship between the microbe and host, but this isn’t going to be simple to assess with pathogens that infect deep tissues and don’t circulate. For example, there aren’t simple methods of determining how many tuberculosis bacilli or pneumonia-causing bacteria are found in an infected lung of a living patient. Likewise, it isn’t simple to determine the amount of hepatitis C virus growing in a liver. These are two simple problems, but there will likely be situations where microbes infect a variety of hard to assay tissues and each tissue will contribute differently to health. Our inability to measure the levels of these pathogens does not mean that these relationships do not exist. We will make progress with systems where it is immediately feasible to do experiments. Success with these infections will drive interest in applying this approach to more difficult situations.

I’ve described these curves as if parasites are simply unchanging particles that need to be counted; this is clearly an oversimplification and we will eventually need to deal with the microbe’s varying contribution to pathology [18]. Hosts and pathogens form systems in which the behavior of each component is so dependent upon the other that we cannot easily separate them. Hosts have tolerance curves and the properties of infecting microbes describe a similar virulence curve. We already know that microbe behavior will change as they find themselves in extracellular or intracellular compartments or within different organs. Microbe behavior will also vary over time as they switch from vegetative to transmissive forms. Still, we can make progress immediately by simplifying the system, and later we can add the complications caused by microbial participation.

## How Can We Define “Health”?

Phase plots like these require microbe loads to be plotted against health, and though it is reasonably clear what constitutes microbe load, defining “health” is more contentious. The answer, from an evolutionary perspective, is that health is the reproductive fitness of an individual. But this isn’t an acceptable answer for modern medicine, where the focus lies on the quality of life and lifespan. In modern medicine, health can be any physiological correlate that matters to patients or scientists. It would be useful to define a core set of standard health measurements, as it would enable us to compare between diseases.

Some commonly used health parameters won’t work for these plots; for example, survival is a terrific health indicator but isn’t useful in a phase plot because it is binary–the host is alive or dead.

To plot phase pictures like those depicted in Figure 3, we need health outputs with a broad dynamic range. It is tempting to use cytokine or immune effector levels as molecular markers, but these don’t necessarily correlate with disease. We know that there is a tolerance curve that correlates health to microbe load. Similarly, for every immune effector or cytokine, there is a tolerance curve that correlates microbe levels to the levels of the effector, but unfortunately there is another tolerance curve that correlates effector level to health. The problem is that we don’t always measure both of these curves. It might be simpler to start with the downstream symptoms that we care about. Gross measurements such as weight, hematocrit, organ function, energy stores, appetite, tissue damage, fever, and activity–disease symptoms–could serve well. Such measurements are often decried as “fuzzy” because we can’t trace directly how these properties connected to the immune response, but this is why these responses are so interesting; that we see individual variation in health in individuals suffering similar infections means that there are processes linking health and immunity that we still don’t understand. The phase picture approach will let us follow these changes and learn what is necessary to help patients; the gross physiological mechanisms that control our daily health are altered by infection.

The idea of health space and disease curves could be used prospectively to find useful health correlates if none exist yet. Imagine an infection where the typical health correlates aren’t providing good indicators of outcome. It might be possible to generate health-by-microbe curves while searching for biomarkers that move in the manner expected for a resilient infection. This approach could be used to identify transcripts or cytokines or metabolites that correlate well with different parts of the expected disease curve, be it the health crash or recovery.

I am using a narrow definition of health; in these plots, “health” measures the current level of some interesting parameter. If that level is normal, then the patient is currently healthy. This could create some confusion with other definitions for health; for example, a patient infected with a single virion of an always fatal virus will appear healthy by these standards but will soon die. Is that person really healthy? This raises the distinction between health as an immediate property and a predictive property; are you healthy now and will you be healthy tomorrow? These curves are currently descriptive and report the path that a patient took through the course of an infection. As we gather a larger data set, the curves will become predictive because we will learn which spaces and velocities suggest trouble. Hopefully we will get to the point where we can look at how health correlates change with respect to each other and predict outcomes without measuring pathogen load directly. No matter how much data we gather, there are some parts of the curves that may never contain much predictive information. The very start of infections could be like this, where the microbe load is too low to measure and health has not yet been knocked out of the normal range.

## Disease Curves Emphasize What We Don’t Know

Perhaps the most important characteristic of these disease curves is that they highlight the parts of disease processes that we have yet to explore. Resilience, the ability to return to the starting state, is one such property. The current focus of host–microbe interactions is on the immune response and subsequent pathogenesis. It is simple to find textbooks concerning the induction of the immune response, immune effectors, or microbial virulence factors. The way we run most experiments makes us experts at describing exactly how sick an individual will get during an infection, but we tend not to measure recovery. Our assumption is that if we can limit the depth of an illness then we will have a shallower hole to escape. We hope to improve patient health by decreasing the distance that a patient has to recover. Does this work?

We know little about how an animal recovers from an infection. Recovery must be an active process. Damage has to be repaired, energy tradeoffs have to be reset, and physiological systems have to be brought back to a standard healthy state. Phase diagrams like those shown in Figure 3 highlight this recovery part of the curve; for example, in Figure 3, curves 2 and 3 (and possibly 4 and 5) show the host is capable of clearing the infection but faces a problem recovering. These curves could provoke questions and new methods for studying recovery. What is the slope of health-by-microbe number in a recovery curve and can this vary? Can recovery occur only after the microbes are cleared or can it start earlier? Do all health traits recover with the same health by microbe slope or health by time slope? Do all pathogenic infection curves trace a clockwise path? These curves would be particularly useful for dissecting processes thought to be involved in the resolution of inflammation or tissue repair.

**Figure 4:**
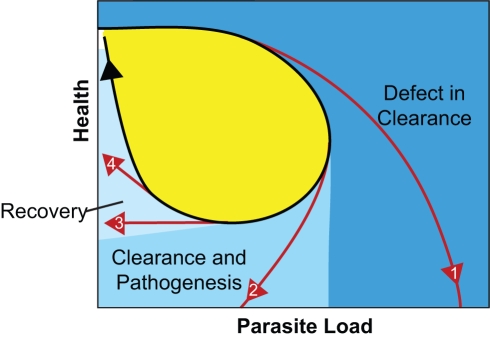
Bifurcation points teach us about defects in the immune response. A resilient disease curve is shown in black and four bifurcating disease curves are shown in red. The first bifurcating curve leads to increased death because of a failure to clear microbes. The second bifurcating curve could have a problem both clearing microbes and preventing pathogenesis. The third and fourth bifurcating curves have defects in recovery but are capable of clearing pathogens. Each bifurcating curve or pair of curves defines regions of disease space that suggest different defects in the immune response.

There are some problems that could particularly benefit from analysis in the phase plane, for example, aging-induced immune senescence and frailty. We know that the immune system changes as we age, but how exactly does this lead to failure? Are disease curves more likely to bifurcate from the normal curves in the aged? If this is the case then we can learn much by determining where the curves are bifurcating. Figure 4 depicts a resilient curve in black and four different bifurcating curves in red. Curve one and other curves like it that would peel off in the dark blue zone would likely have defects in clearing pathogens. Curve two could have defects in both pathogen clearance and damage control. Curves three and four, which peel off in the light blue zone, likely have defects in repair but seem to be able to clear pathogens properly. Does an elderly patient trace the same health space as a young patient but at a different velocity, or is the curve warped? By observing changes in the shape and speed of these curves we could perhaps diagnose the effects of age on immunity better than by studying individual components of the immune response.

## What Do We Need to Do to Make Disease Curves Useful?

If we understood the normal traces of infections, these curves could be immediately helpful to patients in two ways. First, we might be able to define “bad neighborhoods” in the health-by-microbe landscape. These would be regions in which nobody fares well, and when a patient is identified in this region they could be targeted for special attention. With bad neighborhoods, a single sample measuring health and microbe levels would let the physician know whether the patient was at risk. Second, if we could plot a fragment of a patient’s infection curve, we could determine their likely disease trajectory. If the patient has a favorable trajectory they might not need significant support, whereas a patient with a similar general health and microbe load might require immediate assistance. There are situations where it is expected that health will drop, but this could be acceptable so long as the microbes are being cleared at an appropriate rate.

To collect examples of these processes we will have to pick our infections carefully and start with model systems. As discussed above, we should concentrate first on infections where it is easy to quantify the pathogen. However, pathogens that don’t circulate at high levels could be followed using luminescent or fluorescent microbes in model systems by measuring antigen levels in patients or by measuring pathology that was indicative of pathogen load. These experiments obviously have to be done in model organisms that can be infected and repeatedly monitored. If we use small model organisms like flies, worms, and fish that can be ground up and tested for pathogen loads, we can gather large amounts of data. This would remove the personalized disease curve aspect of the process, but if the curves were reproducible and the animals genetically similar this process would still yield the desired information.

Recently there have been arguments made that more work must be done on humans because mechanistic immunological models are not translating well from models into the clinic [27]. One common explanation for this perceived problem is that model organisms are too diverged to teach us about people. A second possibility, highlighted by our lack of understanding of the forces shaping disease curves, is that we have been systematically asking the wrong questions, and we’ve been doing that in at least two different ways: First, we’ve been looking at the immune response without measuring microbes at the same level of resolution (if we measure them at all). The immune response pushes against microbes, and it is impossible to understand the activity of the immune response without knowing what the microbes are doing. Second, we have been looking at proximal immune responses without measuring real health outputs. The precise mechanism of immune activation does not define how sick an animal will get or whether it will be able to recover. We need to connect molecular mechanism with outcome and that has yet to be achieved in any system.
